# Simulation-Based Teaching of Telemedicine for Future Users of Teleconsultation and Tele-Expertise: Feasibility Study

**DOI:** 10.2196/30440

**Published:** 2021-12-22

**Authors:** Benjamin Bouamra, Karim Chakroun, Elisabeth Medeiros De Bustos, Jennifer Dobson, Jeanne-Antide Rouge, Thierry Moulin

**Affiliations:** 1 Department of Neurology Besançon University Hospital Besançon France; 2 Faculty of Medicine University of Franche-Comté MedSim Laboratory Besançon France

**Keywords:** telemedicine, teleconsultation, simulation training, health care, training, education, digital training, medical education

## Abstract

**Background:**

Health care professionals worldwide are increasingly using telemedicine in their daily clinical practice. However, there is still a lack of dedicated education and training even though it is needed to improve the quality of the diverse range of telemedicine activities. Simulation-based training may be a useful tool in telemedicine education and training delivery.

**Objective:**

This study aims to assess the feasibility and acceptability of simulation-based telemedicine training.

**Methods:**

We assessed five telemedicine training sessions conducted in a simulation laboratory. The training was focused on video teleconsultations between a patient and a health care professional. The assessment included the participants’ satisfaction and attitudes toward the training.

**Results:**

We included 29 participants in total. Participant satisfaction was high (mean score 4.9 of 5), and those that took part stated the high applicability of the simulation-based training to their telemedicine practices (mean score 4.6 of 5). They also stated that they intended to use telemedicine in the future (mean score 4.5 of 5).

**Conclusions:**

Simulation-based training of telemedicine dedicated to video teleconsultation was feasible and showed high satisfaction from participants. However, it remains difficult to scale for a high number of health care professionals.

## Introduction

Telemedicine activity has increased considerably worldwide in the past decade. The quality and quantity of telemedicine activities, however, are not yet optimal and could be improved [1]. In France, to improve the quality of telemedicine practices, the Ministry of Health has included telemedicine in the medical curriculum for medical students and residents in recent years [2]. Telemedicine education and training (ET) has also been included in the publicly funded continuing professional development activities available to all physicians. Nevertheless, the proportion of health care professionals (HCPs) trained in telemedicine practice remained limited before the start of the COVID-19 pandemic despite the high demand at the time [3].

Telemedicine ET can involve various components related to clinical guidelines, techniques for remote interactions with the patient, management of a clinical exam performed by another HCP, or the safe and secure use of the technology to support individual and organizational changes [4,5]. The purpose of telemedicine ET may also be to decrease resistance to telemedicine due to potential misconceptions and to increase knowledge and acceptance of the safety and effectiveness of its practice [4].

Simulation-based training has been shown to be an effective method in medical education and should be considered for telemedicine training [6-12]. In simulation-based training, experience creates knowledge that can be transferred to other situations, and failure can be used as leverage in the acquisition of new skills [13]. The objective of the study was to assess the feasibility and acceptability of simulation-based training in telemedicine.

## Methods

### Study Design

We conducted a mixed methods research study, including qualitative analysis of the participants’ perspectives and quantitative assessment of their attitudes toward the training.

### Training Session Design

The training sessions were designed by the Network for Neurological Emergencies at Besançon Regional University Hospital in France and the medical simulation laboratory team at the University of Franche-Comté Medical School. The methodology was based on the guidelines from the *Haute Autorité de Santé* (French National Authority for Health) on simulation-based training in health care [11].

### Training Session Content

**Figure 1:**
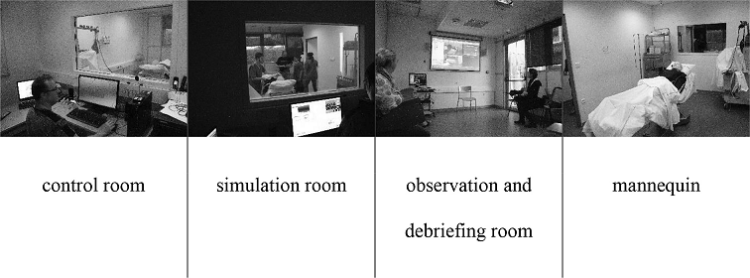
Pictures from the medical simulation laboratory MedSim at the University of Franche Comté Medical School, Besançon, France.

We conducted the training sessions at the simulation laboratory (MedSim) of the University of Franche Comté Medical School. The laboratory has two simulation rooms equipped with cameras, sound systems, and medical equipment, and two observation rooms (used for observation and debrief sessions) equipped with a screen and a projector. Each simulation room is connected to a control room from which medical devices and a simulation mannequin are managed (Figure 1). Trained actors were used to perform scenarios alongside the participants.

Each training session lasted 4 and a half hours. Each session included an introductory component to present the training team, the simulation laboratory, and the program (1 hour). This was followed by a session in which the participants introduced themselves and discussed their initial ideas about telemedicine (1 hour). The training included two scenarios, which both started with a brief (10 minutes), followed by the simulation (20 minutes) and a debrief (30 minutes). The debrief included four steps (description, analysis, application, and summary) and involved discussions between the participants guided by the teaching team. A general debriefing for the whole session (30 minutes) was conducted after the debrief of the second scenario. The training session ended with a survey.

Scenarios were simulations of routine situations, in which the HCPs participated directly or attended as observers. They were designed to provoke discussion about telemedicine as an integrated part of the participants’ routine practices and were not intended to provide technical training on the use of telemedicine.

**Figure 2:**
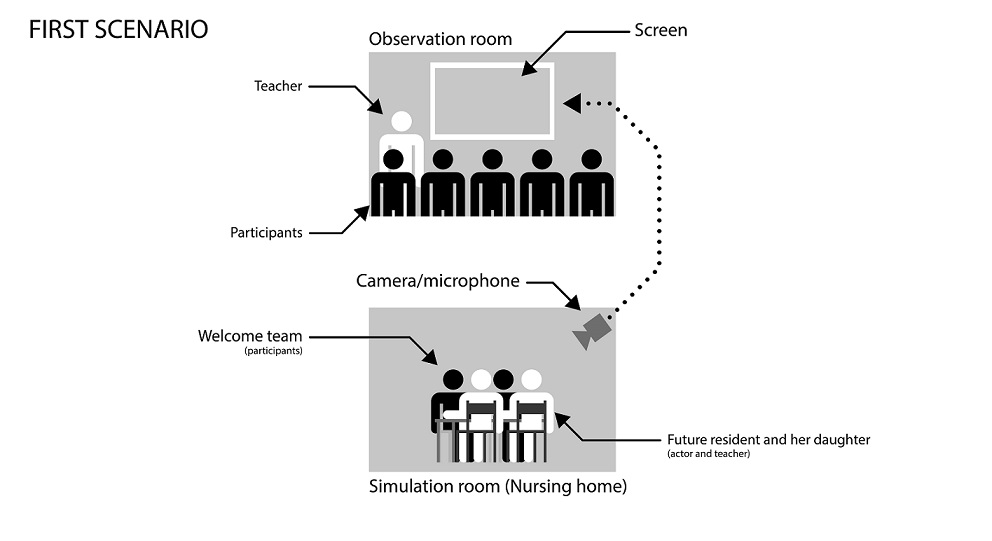
Organization of the first scenario of a telemedicine simulation-based training conducted in the Franche-Comté Region, France between 2018 and 2019.

The first scenario (Textbox 1, Figure 2) focused on understanding what telemedicine is and introduced the ideas of the practical framework, purpose, legal framework, advantages, and limitations of telemedicine. The aim was to reveal the participants’ prior knowledge and attitudes by presenting them with questions that they may be required to answer in routine practice. It was also intended to encourage the participants to seek information on topics such as the differences between a teleconsultation and a physical consultation, legislation, technology, data security, and the medical specialties available via telemedicine in their own health care facilities.

**Figure 3:**
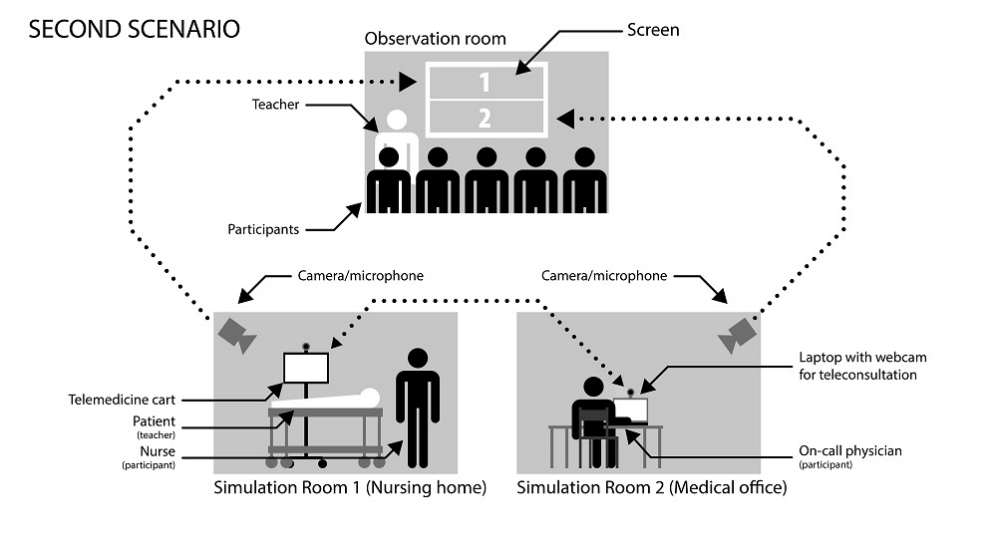
Organization of the second scenario of a telemedicine simulation-based training course conducted in Franche-Comté Region, France between 2018 and 2019.

The second scenario (Textbox 2, Figure 3) was set up to encourage participants to think about the advantages and disadvantages of telemedicine, and how to perform a teleconsultation, guide a clinical examination by telemedicine, direct the participant in repositioning the hardware, and search for clinical signs again if the initial examination was unhelpful.

**The first scenario of a telemedicine simulation-based training course conducted in Franche-Comté Region, France between 2018 and 2019.**

**Simulation room**

The administrative office of a nursing home in which two paramedical professionals (participants) meet with future residents and their families (actors) to answer questions about the home.

**Scenario content**

An older woman with diabetes and no cognitive dysfunction arrives for a meeting at a nursing home with her daughter, who is a retired teacher living in Paris. They are considering transferring the older woman into the nursing home because her daily life has become difficult due to diabetic retinopathy and living alone far from her daughter. The nursing home’s brochure showcases its telemedicine system, and a manager and nurse welcome the visitors and answer their questions concerning the use of telemedicine including:

- “Will I be able to keep seeing my family doctor?”
- “Isn't telemedicine just a cheap substitute for real medicine?”
- “Can I choose whether or not to have teleconsultations?”
- “What about data security and confidentiality?”

**Content of the second scenario of a telemedicine simulation-based training conducted in the Franche-Comté Region, France between 2018 and 2019.**

**Simulation room 1**

The nursing home teleconsultation room contains an examination table and a telemedicine cart (screen linked to a computer with the required telemedicine software and a remote-controlled high-definition camera). The cart and camera are not set up in the ideal orientation for a teleconsultation. The patient (actor) is lying on the examination table. A nurse (participant) has requested the teleconsultation.

**Simulation room 2**

The on-call physician’s office where a consulting physician (participant) has a desk with a laptop computer equipped with the required software and an integrated webcam.

**Scenario content**

An older patient in a nursing home presents with calf pain. The calf is red, hard, and swollen. The patient is disoriented but still able to communicate and has complained of calf pain since that morning. There is no on-site physician and the weather conditions make travel impossible, so the on-call physician (medical professional participant) is contacted to conduct a teleconsultation. The nurse (paramedical professional participant) helps the patient in the teleconsultation room. The physician must conduct the teleconsultation from the office.

### Study Recruitment

Training sessions were scheduled from May 2018 to September 2019 and were publicized by the regional health agency (ARS) to all HCPs in the region who were interested in setting up a telemedicine project. Participants included primary care physicians practicing in nursing homes, hospital geriatricians, health care managers, and nursing home nurses. The training was funded by the ARS of Bourgogne-Franche-Comté.

### Data Collection

The training sessions were conducted by three of the authors (EMDB, BB, JAR). EMDB and BB are practicing physicians (neurologist and emergency physician, respectively) who are experts in telemedicine and simulation training. They have both completed training courses in health care teaching using simulation training as part of their continuing professional development. JAR is a health care manager and is also the head of teaching at the MED-SIM health care simulation training laboratory. She is qualified to train others to teach using simulation.

Two of the three session leaders were present for each training session. For the initial introductory discussion, the leaders asked questions related to the participants’ prior experiences and preconceptions of telemedicine, the telemedicine projects they could become involved in, and their current professional situation. In the debriefing sessions, the leaders used the participants’ feedback and reflections as starting points for guided discussions around the key teaching aims.

Two months prior to the first official session, we conducted a trial session with 4 participants, based on which we made minor changes to the scenarios and structure of the course. In terms of participant recruitment, we accepted all participants who signed up to the training sessions. There was no additional selection process or sampling.

The data were collected at the simulation laboratory. No other participants were present during the data collection. During the discussions, author KC made field notes of the key themes raised by the participants.

A satisfaction survey was conducted at the end of the training with 11 Likert scale questions from 1 (low) to 5 (high) and was a standard questionnaire provided by the MED-SIM simulation laboratory. It has been validated internally by the laboratory and is offered to all participants at all training sessions conducted there. A translation of the items included in the questionnaire is provided in the Results section. We calculated the mean score for each item. Due to the small sample size, we did not conduct any statistical comparisons.

### Ethics Statement

Ethics committee approval was not required for this study in accordance with French legislation. All participants gave their oral consent to the anonymous use of their data for the purpose of this research.

## Results

We conducted five training sessions with an average of 6 participants per session. There were 29 participants in total, including 17 physicians (10 nursing home general practitioners, 4 geriatricians, 2 rehabilitation physicians, and 1 pediatrician in a residential care home) and 12 paramedical professionals (10 nurses and 2 health care managers).

Many of the physicians were initially wary of the idea of performing teleconsultations due to concerns about protecting the patient-physician relationship, constraints of remote clinical examinations, staff being overworked, and payment of private physicians working in nursing homes. The paramedical professionals were less hesitant and mostly had concerns about the distribution of responsibility and logistics.

After the first scenario, participants were particularly interested in data security and had learned that telemedicine is no different to any other tool that could be used to improve the level of care provided for patients. Participants also had a better understanding of telemedicine project implementation methodology, the types of patients who could benefit from telemedicine, and the physical setup of the teleconsultations.

During the second scenario, participants were disoriented by using the technology to communicate. Despite being experienced in physical consultations, the physicians were unsettled and seemed to forget how they would normally communicate with a patient. Their omissions were often remedied during the consultation and the participants noticed the phenomenon of the physicians being disorientated and raised it as a discussion topic during the debrief. The paramedical professionals had the same difficulties, with the addition of being anxious about the technical part of the consultation. Participants noted the limitations of remote communication due to no direct eye contact, lack of visibility and clear understanding of roles of all participants, and delays due to audio/video lags, which can make it difficult to have synchronized conversations.

By the end of the second scenario, the physicians had realized that it was possible to delegate a clinical examination under supervision and to have a high level of confidence in the diagnosis and next patient management steps. Participants also began to imagine themselves in these situations rather than speaking about the concepts in abstract terms. The paramedical professionals showed confidence in clinical examinations supervised by the remote physician.

The results of the survey are presented in Table 1 and the qualitative results are presented in Textbox 3.

**Table 1:** Survey results of a telemedicine simulation-based training conducted in Franche-Comté Region, France between 2018 and 2019.

			Mean score (out of 5)
**Content of the training session**			
	Adherence to the advertised program	4.7	
	Extent to which the teaching content met your expectations	4.7	
	Application to professional practices	4.6	
**Teaching**			
	Quality of teaching	4.9	
	Quality of the materials used	4.8	
	Overall satisfaction	4.9	
	Practical aspects (access to the laboratory, welcome rooms, and equipment)	4.6	
**Impact of the training**			
	I understood the ideas, methods, and techniques that were taught	4.7	
	I intend to put these into practice	4.5

**Sample of comments from the satisfaction questionnaire of the simulation-based training conducted in the Franche-Comté Region, France between 2018 and 2019.**

**Positive comments**

- “Helpful discussions with the session leaders”
- “The course leaders had a lot of practical experience and used very concrete examples”
- “Teaching based on discussions using each person's questions as a starting point”
- “Really relevant debrief sessions”
- “High quality of teaching using real-life examples”
- “Good consideration of the applications and the range of possibilities”

**Negative comments**

- “We would need to train all the staff before putting this into practice”
- “It might be possible to implement this down the line but not immediately”

## Discussion

The telemedicine simulation-based training sessions that were delivered in this study demystified teleconsultations by addressing the participants’ preconceptions and underlying concerns. This study also prioritized a practical understanding of the specificities, benefits, and limitations of telemedicine practice. To our knowledge, this was the first study conducted in France to evaluate simulation for multidisciplinary telehealth training in a broader context than acute telestroke management, which was the sole focus of the previous study [14].

The participants’ feedback regarding the training showed that it reduced their uncertainty toward telemedicine and addressed some of their concerns, particularly for those who had no prior experience of telemedicine. It also enabled them to think about situations in which telemedicine would be helpful and showed that conducting a teleconsultation took them out of their comfort zone and made them forget their usual practices. This is in line with similar results obtained in a study evaluating student nurse practitioners’ perceptions of a simulated gerontological telehealth visit, as well as studies evaluating simulation for telehealth competencies of medical or nursing students [15-17].

The simulation brought to light the fact that telemedicine meets the same criteria as so-called classical medicine (including in terms of medical ethics and patient rights) [18]. In addition to the purely knowledge-based questions about telemedicine from a medical perspective, participants also asked questions about the effectiveness of communication between the different people involved in a teleconsultation such as putting the patient at ease, effectively addressing one of two people, and leading the dialogue to optimize the transmission of information. Practicing communication skills during telehealth visits is also relevant for remotely delivering bad news to the patient or the family [19].

The presence of a telemedicine cart in the room during the first scenario allowed the participants to present the concept clearly and concretely to the future nursing home resident and her daughter. In this scenario, the participants became the *experts* in the tool, and the physical presence of the equipment substantially helped them with their explanations [20].

The main limitations of this teaching method are time and financial cost (eg, number of staff required, preparation, and testing of different scenarios), equipment needed, and the low number of participants per session, which is necessary to maximize the impact. Technical issues may also create difficulties. A simulation laboratory necessarily involves the installation and maintenance of multimedia devices and their connections. Additionally, telemedicine training sessions require specific devices, which further complicates the different interfaces. The number of sessions that it is possible to run is therefore also limited by the availability of people with the required technical and human competences.

Due to the rapid increase of telemedicine during the course of the pandemic, in situ simulation for telemedicine training directly from the health care settings may be a more practical and cost-effective method than using a simulation lab [21]. Simulating video teleconsultations through easily accessible software may also be a pragmatic approach to large-scale telehealth training [22].

The artificial nature of communication during teleconsultations requires specific training, which could be delivered by an expert in communication and public speaking. This could lead to the development of a teleconsultation protocol, which could improve communication by avoiding misunderstandings and hesitations, both between the HCPs and with the patient.

Until now, HCPs who are motivated and involved in setting up telemedicine projects have learned this new way of medical practice through experience. As the use of telemedicine becomes more widespread, users who have no prior experience need to integrate these concepts into their practices. In this context, *on-the-job* learning is less desirable.

It therefore seems necessary to establish practical training frameworks with the principal aim of allowing participants to structure telemedicine practices, especially teleconsultation practices, in the most effective way possible. Learning by simulation would seem to be the most appropriate solution. This type of approach is being developed in the United Kingdom, where simulation training is being used to teach webside manner to medical students for video teleconsultations [23]. Simulation-based teaching of telemedicine is an immersive and practical approach that is particularly suited to the continuing professional development of medical and paramedical health care personnel who are involved in an operational telemedicine project. This training could complement an Inter University Diploma in Telemedicine, which primarily caters to people who are setting up a telemedicine project and requires a greater level of personal investment.

The effectiveness of simulation-based learning in health care is well documented [8,9]. In addition to skills, simulation can be used to teach practical techniques to medical and paramedical personnel [24]. According to Policard [25], experience-based learning and reflecting on practices “help to create a shared vision of the situation/problem, as well as common operational frameworks that aid communication and promote teamwork.” Simulation exercises also facilitate teamwork and the implementation of procedures, which has a positive impact on patients [26,27]. Simulation-based training would therefore meet the needs of HCPs by providing a pragmatic approach and concrete examples of what a telemedicine act looks like in practice. However, the cost of this means that it may only be a short-term solution. Ultimately, relevant university teaching curricula should integrate telemedicine training [28].

## Abbreviations

ARS: regional health authority
ET: education and training
HCP: health care professional
